# Novel Heme Oxygenase-1 Inducers Palliate Inflammatory Pain and Emotional Disorders by Regulating NLRP3 Inflammasome and Activating the Antioxidant Pathway

**DOI:** 10.3390/antiox12101794

**Published:** 2023-09-23

**Authors:** Montse Pérez-Fernández, Irene Suárez-Rojas, Xue Bai, Ignacio Martínez-Martel, Valeria Ciaffaglione, Valeria Pittalà, Loredana Salerno, Olga Pol

**Affiliations:** 1 Grup de Neurofarmacologia Molecular, Institut d’Investigació Biomèdica Sant Pau (IIB Sant Pau), 08041 Barcelona, Spain; 2 Grup de Neurofarmacologia Molecular, Institut de Neurociències, Universitat Autònoma de Barcelona, 08193 Barcelona, Spain; 3 Institute of Crystallography, National Council of Research (CNR), 95126 Catania, Italy; 4 Department of Drug and Health Sciences, University of Catania, 95125 Catania, Italy

**Keywords:** analgesia, anxiety, depression, heme oxygenase-1, inflammasome, inflammatory pain, Nrf2 transcription factor, oxidative stress

## Abstract

Chronic pain caused by persistent inflammation is current in multiple diseases and has a strong negative impact on society. It is commonly associated with several mental illnesses, which can exert a negative influence on pain perception, and needs to be eradicated. Nevertheless, actual therapies are not sufficiently safe and effective. Recent reports demonstrate that the induction of heme oxygenase-1 (HO-1) enzyme produces analgesic effects in animals with osteoarthritis pain and reverses the grip strength loss caused by sciatic nerve crush. In this research, we evaluated the potential use of three new HO-1 inducers, 1m, 1a, and 1b, as well as dimethyl fumarate (DMF), for treating persistent inflammatory pain induced by the subplantar injection of complete Freud’s adjuvant and the functional deficits and emotional sickness associated. The modulator role of these treatments on the inflammatory and antioxidant pathways were also assessed. Our findings revealed that repeated treatment, for four days, with 1m, 1a, 1b, or DMF inhibited inflammatory pain, reversed grip strength deficits, and reversed the linked anxious- and depressive-like behaviors, with 1m being the most effective. These treatments also suppressed the up-regulation of the inflammasome NLRP3 and activated the expression of the Nrf2 transcription factor and the HO-1 and superoxide dismutase 1 enzymes in the paw and/or amygdala, thus revealing the anti-inflammatory and antioxidant capacity of these compounds during inflammatory pain. Results suggest the use of 1m, 1a, 1b, and DMF, particularly 1m, as promising therapies for inflammatory pain and the accompanying functional disabilities and emotional diseases.

## 1. Introduction

Chronic pain is defined by the International Association of the Study of Pain as “an unpleasant sensory and emotional experience associated with actual or potential tissue damage or described in terms of such damage” [1]. Chronic pain is a complex disease highly manifested in the adult population, being an important social and public health problem with a strong impact on society [2]. Chronic pain is related to an increased sensitivity to painful stimulus (hyperalgesia) and the perception of pain after a non-painful stimulus (allodynia), and also includes a maladjustment in physical functioning that negatively affects patients with inflammatory diseases. Therefore, and due to the positive correlation between pain and physical functioning, an evaluation of the effects of treatments on physical function is highly recommended [3]. In addition, it is well recognized that chronic pain is associated with significant emotional disorders, such as anxiety and/or depression, which are not only related to the development of chronic pain, but can also increase the sensitivity to pain [4,5]. The current approaches to palliate chronic inflammatory pain mainly rely on the use of opioids and non-steroidal anti-inflammatory drugs (NSAIDs). However, despite all efforts, these therapies are not fully effective for treating the functional and affective disorders linked with inflammatory pain, and, further, they carry important side effects [6]. Therefore, further investigations are necessary for developing better treatments for chronic inflammatory pain.

It has been known that, during the inflammatory process, the production of mitochondrial reactive oxygen species (ROS) can be potentiated. The increase in ROS induces the activation of several inflammasomes, especially NLRP3 [7]. This inflammasome consists of the sensor molecule NOD-like receptor protein 3 (NLRP3), the apoptosis-associated speck-like protein (ASC), and a precursor caspase-1 (pro-caspase-1). This complex triggers the maturation of several pro-inflammatory cytokines, such as interleukin-1β (IL-1β) and IL-18 [7]. Consequently, the NLRP3 activation is related to several inflammatory diseases, for instance inflammatory pain, and with the depressive-like behaviors provoked by lipopolysaccharides [8,9]. Therefore, the inactivation of NLRP3 inflammasome could be a strategy for treating inflammatory pain.

Nuclear factor (erythroid-derived 2)-like 2 (Nrf2) is a transcriptional factor that plays a crucial role in the regulation of many antioxidant, anti-inflammatory, and cell survival genes [10]. Its activation inhibits chronic pain, including inflammatory and neuropathic [11,12,13]. These effects are mainly related to the fact that, under normal conditions, Nrf2 binds to Kelch-like ECH associated-protein 1 (Keap1) in the cytoplasm and forms the Nrf2-Keap1 complex. However, under oxidative stress situations, it is dissociated and the Nrf2 migrates to the nucleus and initiates the transcription of multiple genes related to the antioxidant responses, such as heme oxygenase-1 (HO-1) or NAD(P)H: quinone oxidoreductase 1 (NQO1), glutathione S-transferase (GSTM1), and superoxide dismutase 1 (SOD-1), among others [14]. Consequently, targeting this pathway can be beneficial for the treatment of chronic pain.

There are several types of Nrf2 activators; one of them is dimethyl fumarate (DMF), a derivate of fumaric acid [15] that is approved by the FDA for the treatment of multiple sclerosis [16]. DMF has multiple properties, for instance anti-inflammatory, antioxidant, and neuroprotective properties [17]. DMF exerts a positive impact on the antioxidant system by modifying Keap-1 and preventing its binding to Nrf2, thus permitting that Nrf2 to be translocated into the nucleus and activating the antioxidant genes’ transcription [18]. The analgesic properties of DMF in animals with osteoarthritis [19,20], neuropathic [21], and post-operative pain [22] have been recently revealed. It also has been proven that DMF improves the grip strength deficits observed after a crush of the sciatic nerve [23] and that produced by multiple sclerosis [24]. Furthermore, it has been seen that DMF attenuates gouty arthritis and colitis by suppressing NLRP3 inflammasome activation [25,26]. However, the effects of DMF on the expression of NLRP3 from animals with allodynia, hyperalgesia, and grip strength deficits provoked by a peripheral inflammation have not yet been assessed.

One of the principal genes regulated by Nrf2 is HO-1, a potent cytoprotective and anti-inflammatory enzyme that degrades heme into carbon monoxide, free iron, biliverdin, and bilirubin. In mammals, three isoforms of HO had been discovered. HO-1 is an isoform that is induced during different pathophysiology conditions and has anti-inflammatory and antinociceptive effects, in part by decreasing the expression of pro-inflammatory cytokines (IL-6, IL-1 and TNF-α) [27]. On the contrary, the HO-2 isoform has been more associated with pronociceptive properties [28] and HO-3 is still an elusive and poorly understood isoform.

**Figure 1:**
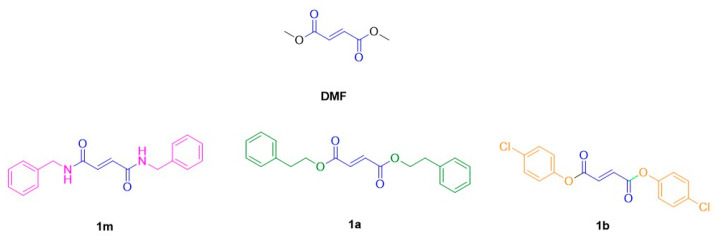
Chemical structure of novel HO-1 inducers based on the DMF structure, 1m, 1b, and 1a, synthetized by [29,30].

Recently, the new DMF derivatives 1m, 1a, and 1b (Figure 1) were synthesized at the University of Catania with the objective of producing more potent HO-1 inducers [29,30]. Each of these derivatives was designed maintaining the α, β-unsaturated dicarbonyl function of DMF as the central chain crucial for HO-1 induction (blue) and introducing different symmetric substituted or unsubstituted phenyl rings by means of an ester or amide linkage. In particular, compound 1m carried an unsubstituted benzyl amide moiety (magenta), whereas 1a contained a phenethyl ester (green), and 1b a 4-chloro phenyl ester (orange).

In these studies, the authors demonstrated in vitro that 1b and 1m both increase the HO-1 expression even more effectively than DMF, and 1m is the most potent compound in increasing HO-1 activation. The authors also proved that these molecules are devoid of cytotoxic activity and can reduce ROS production caused by palmitic acid. However, their possible antinociceptive, anxiolytic, and/or antidepressant effects during inflammatory pain and the mechanisms implied are unidentified.

Therefore, in a male mice model of complete Freund’s adjuvant (CFA)-induced inflammatory pain, our objectives are to evaluate the effects of treatment with DMF, 1m, 1a, and 1b, on: (1) the mechanical allodynia and thermal hyperalgesia provoked by peripheral inflammation; (2) the grip strength deficits caused by paw inflammation; (3) the anxiety and depressive-like behaviors related to persistent inflammatory pain; and (4) the expression of NLRP3 inflammasome and the antioxidant proteins Nrf2, HO-1, SOD-1, NQO1, and GSTM1 in paw tissues and amygdala.

## 2. Materials and Method

### 2.1. Animals

Male C57BL/6 mice purchased from Envigo Laboratories (Barcelona, Spain) were used. These animals were under 12/12 h light/dark conditions and controlled temperature (22 ± 1 °C) and humidity (55 ± 10%) with able access to food and water. The experiments were carried out after 7 days of acclimatation to the housing conditions and between 9:00 a.m. and 5:00 p.m. In accordance with the guidelines stablished by the European Commission’s directive and Spanish Law (RD 53/2013) and approved by the local Committee of Animal Use and Care of the Autonomous University of Barcelona (ethical code 9863), every attempt was made to minimize the suffering and the number of animals used.

### 2.2. Generation of Inflammatory Pain

To induce the inflammatory pain, animals were subplantarly injected with 30 µL of CFA (Sigma-Aldrich, St. Louis, MO, USA) in the right hind paw. This process was performed under short anesthetic conditions with isoflurane (3% induction and 3% maintenance) [11].

### 2.3. Allodynia, Hyperalgesia, and Grip Strength Measurements

Mechanical allodynia was assessed using the von Frey filaments. Mice were located on a wire grid through where the von Frey filaments of different strength (North Coast Medical, Inc., San Jose, CA, USA) were applied following the up–down paradigm proposed by [31]. The filaments had between 0.4 to 3.0 g of bending force. Withdrawing, shaking, or licking the hind paw was considered a positive reaction. Based on the animal’s answer to a filament, the following filament applied was of higher or lower strength.

Thermal hyperalgesia was evaluated using the plantar test (Ugo Basile, Varese, Italy) as described by [32]. The mice were positioned on a glass surface inside Plexiglas tubes (20 cm high × 9 cm diameter). The heat source was applied under the hind paws. The paw withdrawal latencies were obtained from the mean of three separate trials. To prevent paw tissue damage, mice were exposed to the head for a maximum of 12 s.

The hind paws grip strength was measured using a computerized grip strength meter (Model 47200. Ugo Basile, Varese, Italy) [33]. The animal had to grasp with both hind legs a metal bar connected to the grip strength meter that recorded the maximum force of each measurement (g). Between 3 to 4 measures were performed for each animal.

### 2.4. Emotional-like Behaviors

The assessment of the anxiety-like behaviors was conducted using the elevated plus maze (EPM) [34] and open field (OF) [35] tests.

In the EPM, we used a maze that was elevated at a height of 45 cm from the floor and consisted of four arms, two closed and two opened. The closed arms were enclosed by walls measuring 15 cm. In the initiation of the test, mice were placed in the central square of the maze, always facing one open arm. We recorded the mouse’s behavior for 5 min, allowing, and quantified the number of entries into the close and open arms and the percentage of time spent in the open arms.

The second test conducted to evaluate anxiety-like behaviors was the OF, which consisted of putting the animal in the center of a square box measuring 44 cm × 44 cm, with walls standing at a height of 30 cm. They were allowed to explore the box freely for 5 min and their behavior was recorded. Then, the number of squares crossed and entries into the central area, and the time passed in it, were quantified.

To assess the depressive-like behaviors of the animals, we performed the tail suspension test (TST) [36] and the force swimming test (FST) [37].

In the FST, mice were suspended from an elevated surface of approximately 35 cm from the ground. The animal was attached to the surface with an adhesive tape attached to the tail. We recorded 8 min for each animal and measured its immobility time from the last 6 min.

In the FST test, the animals were collocated in a cylinder with 10 cm of water at 24 ± 1 °C. Each animal was recorded for 6 min, and its inactivity time was gauged for the last 4 min.

All these experiments were performed by investigators blinded to the experimental conditions and animals were habituated to the environment for 1 h before the experiment.

### 2.5. Western Blot

Mice were euthanized by cervical dislocation at 16 days after CFA or saline solution (SS) injection. The ipsilateral subplantar paw and the contralateral amygdala were dissected and quickly preserved at −80 °C for later analysis. To evaluate the levels of proteins NLRP3, Nrf2, HO-1, SOD-1, NQO1, and GSTM1, the extracted tissues were homogenized using an ice-cold lysis buffer consisting of RIPA buffer, 0.5% protease inhibitor cocktail, and 1% phosphatase inhibitor cocktail (Sigma-Aldrich, St. Louis, MO, USA). The homogenate tissues stayed at 4 °C for 1 h, after which they were sonicated (10 s) and centrifugated at 4 °C for 20 min at 1000 rpm. The supernatant (60 µg of total protein) was mixed with 4× Laemmli loading buffer and loaded onto a 12% separating sodium dodecyl sulphate polyacrylamide gel. Once the electrophoresis was done, proteins were transferred onto a polyvinylidene fluoride membrane for 120 min. Membranes were blocked with phosphate-buffered saline plus Tween 20 or Tris-buffered saline plus Tween 20 and different percentages of non-fat dry milk or bovine serum albumin. After 75 min, the blocking solution was discarded, and membranes were incubated overnight at 4 °C with a specific primary antibody, anti NLRP3 (1:200; Adipogen Life Sciences, Epalinges, Switzerland); Nrf2 (1:115; Abcam, Cambridge, UK); HO-1 (1:100; Enzo Life Science, New York, NY, USA); SOD-1 (1:150; Novus Biologic, Littleton, CO, USA); NQO1 (1:200; Sigma-Aldrich, St. Louis, MO, USA); or GSTM1 (1:100; Novus Biologic, Littleton, CO, USA), and anti-glyceraldehyde-3-phosphate dehydrogenase (GAPDH, 1:5000; Merck, Billerica, MA, USA) as a loading control. Then, membranes were incubated with a blocking solution together with horseradish peroxidase-conjugated anti-rabbit or anti-mouse secondary antibodies for 1 h at room temperature.

Furthermore, using ChemiDoc and chemiluminescent reagents, proteins were detected. After capturing an image of the membrane, each protein band was analyzed by densitometry employing the Image-J program (National Institutes of Health, Bethesda, MD, USA).

### 2.6. Drugs

DMF (Sigma–Aldrich, St. Louis, MO, USA) and 1m, 1b, and 1a synthesized by [29,30] were dissolved in 0.05% of Cremophor EL (CMC)/dimethyl sulfoxide/ethanol (Sigma-Aldrich, St. Louis, MO, USA) in a mixture of 12:2:2 in accordance with another study [21]. All drugs were prepared immediately before use in a final volume of 10 mL/kg. For each group injected with a drug, the corresponding control group was given an identical volume of vehicle (0.05% CMC/dimethyl sulfoxide/ethanol in a mixture of 12:2:2; VEH).

### 2.7. Experimental Design

In the first experiment, at 13 days of SS or CFA injection, mice were administered with 300 mg/kg of DMF, 1m, 1a, 1b, or VEH given orally for 4 consecutive days. In these animals, we assessed the mechanical allodynia, thermal hyperalgesia, and grip strength at day 0 (before SS or CFA injection), at day 12 (pre-treatment), and every day of treatment until day 16 after SS or CFA injection, after 3 h of drug administration. We used 6 animals for each group of SS-injected mice (SS-VEH, SS-DMF, SS-1m, SS-1a, and SS-1b) and 6 animals for each group of CFA-injected mice (CFA-VEH, CFA-DMF, CFA-1m, CFA-1a, and CFA-1b), 60 mice in total.

In other groups of animals, we studied the anxiolytic and antidepressant effects produced by the same drugs, DMF, 1m, 1a, and 1b, also orally administered, at 300 mg/kg, during 4 consecutive days (from day 13 to 16 after CFA injection). Tests were performed on the last day of administration (day 16) and at 3 h after the oral administration of DMF, 1m, 1a, and 1b. We used 8 animals per group (SS-VEH, CFA-VEH, CFA-DMF, CFA-1m, CFA-1a, and CFA-1b); that is a total of 48 mice. In total, 108 mice were used.

Lastly, the protein levels of NLRP3, Nrf2, HO-1, SOD-1, NQO1, and GSTM1 in the paw and amygdala of CFA-injected animals treated with DMF, 1m, 1a, and 1b were analyzed. Controls were SS-injected mice treated with VEH (n = 3 samples per group).

### 2.8. Statistical Analyses

The GraphPad Prism 9.0 software (La Jolla, CA, USA) and SPSS version 28 (IBM, Madrid, Spain) were employed for the statistical analysis. The normal distribution of the data was determined using the Kolmogorov–Smirnov test. A three-way analysis of variance (ANOVA) with repeated measures was used to evaluate the effects of inflammation, treatment, and time of treatment and their possible interactions in animals treated with DMF, 1m, 1a, and 1b or VEH. For each day of treatment, the possible differences between groups were evaluated by using a one-way ANOVA followed by the Tukey post-test.

The one-way and Tukey post-test was also employed to evaluate the possible differences between DMF, 1m, 1a, and 1b or VEH treatments on the anxiety- and depressive-like behaviors as well as in their effects on the expression of different proteins in the paw and amygdala.

In all cases, *p* < 0.05 was considered statistically significant. Results are plotted as the mean ± standard error of the mean (SEM).

## 3. Results

### 3.1. Effects of the Treatment with DMF, 1m, 1a, and 1b on the Mechanical Allodynia, Thermal Hyperalgesia, and Grip Strength Loss Caused by CFA

**Figure 2:**
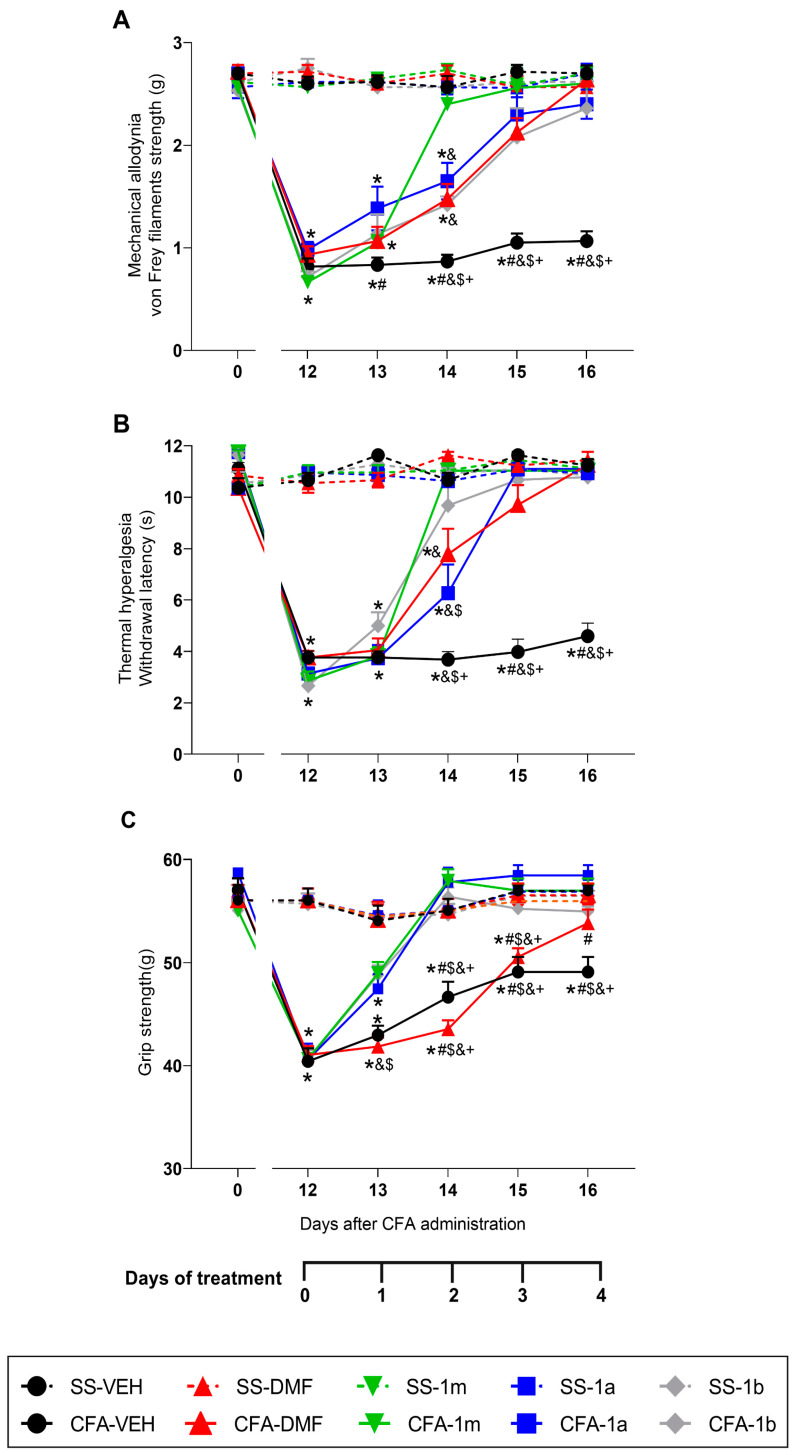
The inhibition of the mechanical allodynia, thermal hyperalgesia, and grip strength deficits provoked by CFA with the repetitive administration of DMF, 1m, 1a, and 1b. Effects of the repetitive administration of DMF, 1m, 1a, 1b, or VEH on the reduced von Frey filaments strength (g) (**A**), withdrawal latency (s) (**B**) and grip strength (g) (**C**) generated by CFA in the ipsilateral hind paws. For each test, symbols represent significant differences vs. * their respective SS group, + CFA plus DMF treated animals, & CFA plus 1m treated animals, $ CFA plus 1b treated animals, and # CFA plus 1a treated animals (*p* < 0.05, one-way ANOVA followed by Tuckey test). Data are presented as mean values ± SEM; n = 6 animals for group.

We evaluated the effects of the administration of DMF, 1m, 1a, and 1b, for four repetitive days, on the mechanical allodynia, thermal hyperalgesia, and grip strength deficits induced by peripheral inflammation from day 13 to day 16 after CFA injection (Figure 2).

In the case of mechanical allodynia, the three-way repeated measures ANOVA revealed significant effects of inflammation (*p* < 0.003), time (*p* < 0.000), and treatment (*p* < 0.05). It also showed significant interactions between inflammation and treatment (*p* < 0.05), inflammation and time (*p* < 0.000), and treatment and time (*p* < 0.001) and a triple interaction between inflammation, treatment, and time (*p* < 0.000). The results indicated that the decreased threshold of the ipsilateral hind paw withdrawal to von Frey filaments stimulus caused by CFA (*p* < 0.001, one-way ANOVA vs. SS-injected mice treated with VEH) was completely reverted after two days of treatment with 1m and these effects were higher than those produced by DMF, 1a, and 1b at the same day (*p* < 0.001, one-way ANOVA) (Figure 2A). Moreover, three days of treatment with DMF, 1a, and 1b were required to block the allodynia.

For thermal hyperalgesia, the three-way repeated measures ANOVA revealed significant effects of inflammation (*p* < 0.000), treatment (*p* < 0.000), and time (*p* < 0.000) as well as interactions between inflammation and treatment (*p* < 0.000), inflammation and time (*p* < 0.000), treatment and time (*p* < 0.000), and between inflammation, treatment, and time (*p* < 0.000) (Figure 2B). In this case, while only two days of treatment with 1m and 1b were required to completely reverse the reduced paw withdrawal provoked by CFA (*p* < 0.001, one-way ANOVA vs. SS-injected mice treated with VEH), three days of treatment with DMF and 1a were necessary. Our results also revealed that, at two days of treatment, the antihyperalgesic effects produced by 1 m and 1b were higher than those produced by DMF and/or 1a at the same day.

Concerning the grip strength deficits provoked by CFA, the three-way repeated measures ANOVA revealed significant effects of inflammation (*p* < 0.027), treatment (*p* < 0.001), and time (*p* < 0.000). It also showed significant interactions among inflammation and treatment (*p* < 0.002), inflammation and time (*p* < 0.000), treatment and time (*p* < 0.000), and between the three of them (*p* < 0.000). After a strong loss of grip strength at 12 days from CFA injection, a similar recovery in VEH- and DMF-treated mice was observed in the following 3 days, but, at 16 days post-CFA injection, the recovery of grip strength in DMF-treated mice was significantly improved compared with VEH-treated mice (*p* < 0.001, one-way ANOVA) (Figure 2C). In contrast, a completed reversion of the grip strength deficits induced by CFA (*p* < 0.001, one-way ANOVA vs. SS-injected mice treated with VEH) was observed after two days of treatment with 1m, 1a, and 1b.

In all tests, no significant effects of the repetitive administration of DMF, 1m, 1a, and 1b were detected in the ipsilateral paws of SS-injected mice (Figure 2), nor in the contralateral paws of CFA- or SS-injected mice.

### 3.2. Effects of Treatment with DMF, 1m, 1a, and 1b on the Anxiety-like Behaviours Associated with Inflammatory Pain

**Figure 3:**
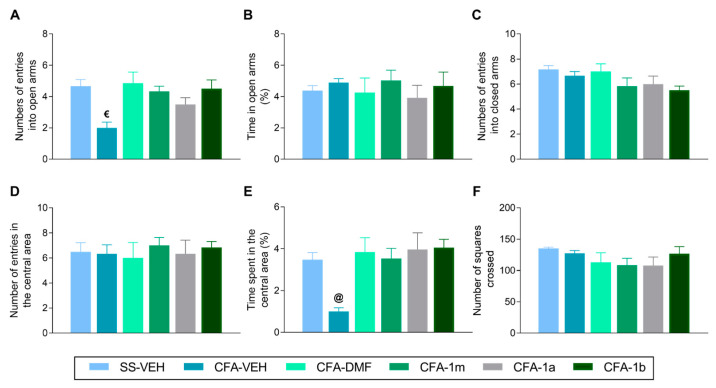
Inhibition of anxiety-like behaviors induced by CFA with the repetitive administration of DMF, 1m, 1a, and 1b. The effects of the oral administration of DMF, 1m, 1a, and 1b in the EPM and OF tests, during four consecutive days, at 16 days after CFA injection, are shown. The behaviors evaluated in the EPM are the number of entries into open arms (**A**), the percentage of time spent (**B**), and the number of entries into closed arms (**C**). In the OF test, the number of entries in the central area (**D**), the percentage of time spent in the central area (**E**), and the number of squares crossed (**F**) are represented. The effects of VEH in SS-injected mice are also displayed. For each test evaluated, different symbols represent significant differences vs.: € all the groups except CFA-injected mice treated with 1a, and @ the rest of groups, (*p* < 0.05; one-way ANOVA and Tukey test). Data are presented as mean values ± SEM; n = 8 animals for group.

To study if repetitive oral administration with DMF, 1m, 1b, and 1a for four days can inhibit the anxiety-like behaviors accompanying inflammatory pain, we used the EPM and TST. The results obtained from the EPM showed that the decreased number of entries into the open arms observed in CFA-plus-VEH-treated mice as compared with SS–VEH-treated animals (*p* < 0.003, one-way ANOVA) (Figure 3A) was reversed with DMF, 1m, 1a, and 1b treatments (Figure 3A). No changes between groups were observed, nor in the amount of time that the animals spent in the open arms (Figure 3B), nor in the number of entries into the closed arms (Figure 3C).

Regarding the OF test, data also revealed that the decreased percentage of time that CFA-injected animals treated with VEH spent in the central area (*p* < 0.000, one-way ANOVA vs. SS-plus-VEH-treated mice) was normalized with the four treatments (Figure 3E). No alterations between groups were detected, nor in the number of entries into the central area (Figure 3D) or in the number of squares crossed (Figure 3F).

### 3.3. Effects of Treatment with DMF, 1m, 1a, and 1b on the Depressive-like Behaviours Linked with Inflammatory Pain

**Figure 4:**
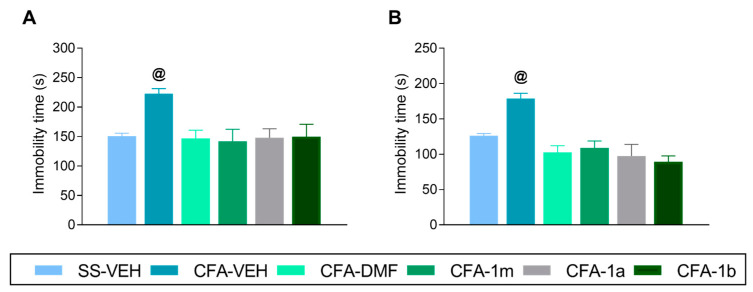
Inhibition of the depressive-like behaviors accompanying inflammatory pain with the repetitive administration of DMF, 1m, 1a, and 1b. The effects of the oral administration of DMF, 1m, 1a, and 1b in the TST and FST during four consecutive days, at 16 days after CFA injection, are shown. Pannels (**A**,**B**) represent the immobility time(s) in the TST and FST, respectively. The effects of VEH in SS-injected animals are also displayed. For each test, @ represents significant differences vs. the rest of the groups (*p* < 0.05, one-way ANOVA and Tuckey test). Data are shown as mean values ± SEM; n = 8 animals for group.

To study if repetitive oral administration with DMF, 1m, 1a, and 1b, for four days, could inhibit the depressive-like behaviors accompanying inflammatory pain, we utilized the TST (Figure 4A) and FST (Figure 4B). In both tests, the increased immobility time caused by CFA (*p* < 0.005, one-way ANOVA vs. their corresponding SS-plus-VEH-treated animals) was inhibited after four days of treatment with DMF, 1m, 1a, or 1b.

### 3.4. Effects of DMF, 1m, 1a, and 1b Treatments on the Expression of NLRP3, Nrf2, HO-1, SOD-1, NQO1, and GSTM1 in the Paw and Amygdala from CFA-Injected Mice

The actions of the repetitive administration of DMF, 1m, 1a, and 1b during four consecutive days on the levels of protein related to the inflammatory (NLRP3) and antioxidant (Nrf2, HO-1, SOD-1, NQO1, and GSTM1) pathways were evaluated.

**Figure 5:**
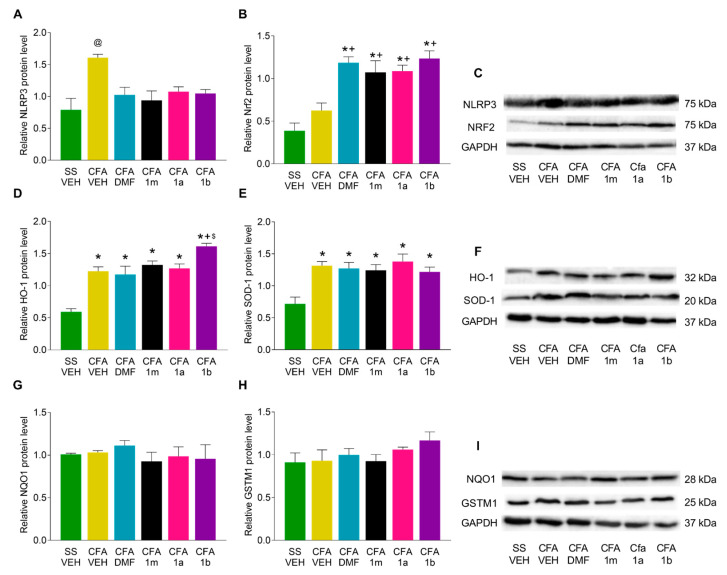
Effects of DMF, 1m, 1a, and 1b treatments on the expression of NLRP3, Nrf2, HO-1, SOD-1, NQO1, and GSTM1 in the paw tissues from animals with peripheral inflammation induced by CFA. The protein levels of NLRP3 (**A**), Nrf2 (**B**), HO-1 (**D**), SOD-1 (**E**), NQO1 (**G**), and GSTM1 (**H**) in the ipsilateral paw of CFA-injected mice treated during four consecutive days with DMF, 1m, 1a, 1b, or VEH are represented. SS-injected mice treated with VEH are also shown. In all graphics, symbols indicate significant differences: @ vs. the rest of groups, * vs. SS mice treated with VEH, + vs. CFA-injected mice treated with VEH, and $ vs. CFA-injected mice treated with DMF (*p* < 0.05; one-way ANOVA and Tuckey test). Representative examples of blots for NLRP3 and Nrf2 (**C**), HO-1 and SOD-1 (**F**), and for NQO1 and GSTM1 proteins (**I**), in which GAPDH was used as a loading control, are displayed. Data are shown as the mean ± SEM; n = 3 samples per group.

In the paw tissues of CFA-injected mice, results indicated that the over-expression of the NLRP3 inflammasome (*p* < 0.002, one-way ANOVA vs. SS plus VEH) was normalized with the four assessed treatments (Figure 5A). It was also seen that the administration of DMF, 1m, 1a, and 1b significantly enhanced the expression of Nrf2 when compared with animals with and without peripheral inflammation treated with VEH (*p* < 0.000, one-way ANOVA) (Figure 5B). Our results also showed that the protein levels of HO-1 (Figure 5D) were significantly up-regulated in CFA-injected animals treated with VEH (*p* < 0.000, one-way ANOVA vs. SS-plus-VEH-treated animals), this high expression was preserved after treatment with DMF, 1m, and 1a, and even HO-1 levels were additionally amplified in mice receiving 1b as compared with those receiving VEH or DMF (*p* < 0.000, one-way ANOVA). In the case of SOD-1, data revealed that its expression increased in CFA-injected animals treated with VEH, DMF, 1m, 1a, or 1b (*p* < 0.001, one-way ANOVA vs. SS animals treated with VEH) (Figure 5E). No significant differences in the expression of NQO1 (Figure 5G) or GSTM1 (Figure 5H) were detected between the different analyzed groups.

**Figure 6:**
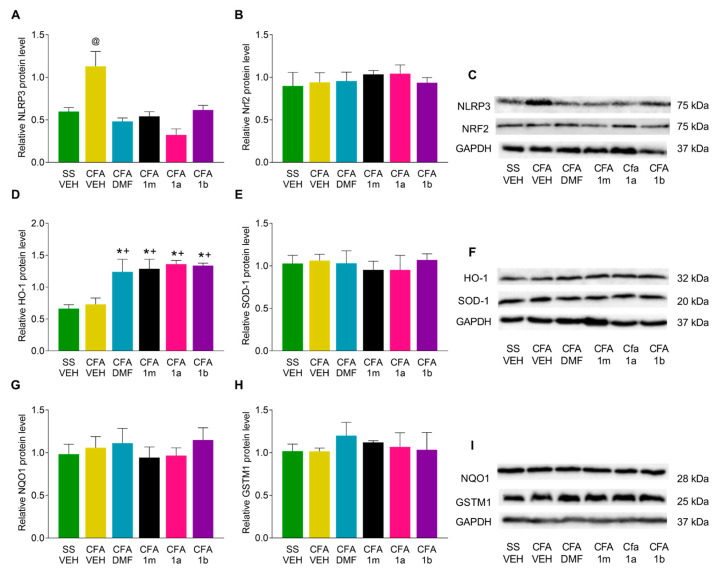
Effects of DMF, 1m, 1a, and 1b treatments on the expression of NLRP3, Nrf2, HO-1, SOD-1, NQO1, and GSTM1 in the amygdala from animals with peripheral inflammation induced by CFA. The protein levels of NLRP3 (**A**), Nrf2 (**B**), HO-1 (**D**), SOD-1 (**E**), NQO1 (**G**), and GSTM1 (**H**) in the amygdala of CFA-injected mice treated during four consecutive days with DMF, 1m, 1a, 1b, or VEH are represented. SS-injected mice treated with VEH are also shown. In all graphics, different symbols indicate significant differences: @ vs. the rest of groups, * vs. SS-injected mice treated with VEH and + vs. CFA-injected mice treated with VEH (*p* < 0.05; one-way ANOVA and Tuckey test). Representative examples of blots for NLRP3 and Nrf2 (**C**), HO-1 and SOD-1 (**F**), and for NQO1 and GSTM1 proteins (**I**), in which GAPDH was used as a loading control, are displayed. Data are shown as the mean ± SEM; n = 3 samples per group.

The expression of NLRP3, Nrf2, HO-1, SOD-1, NQO1, and GSTM1 were also evaluated in the amygdala of CFA-injected animals treated during four days with DMF, 1m, 1a, 1b, or VEH. In this case, the up-regulation of the NLRP3 inflammasome provoked by CFA (*p* < 0.000, one-way ANOVA vs. SS animals treated with VEH) was normalized with the treatment with DMF, 1m, 1a, and 1b (Figure 6A). In this tissue, we also observed that all treatments significantly increased the expression of HO-1 (*p* < 0.000, one-way ANOVA vs. SS- or CFA-plus-VEH-treated mice) (Figure 6D). Non-differences in the expression of Nrf2 (Figure 6B), SOD-1 (Figure 6E), NQO1 (Figure 6G), or GSTM1 (Figure 6H) were found between the studied groups.

## 4. Discussion

This study demonstrates the analgesic effects and reversal of grip strength deficits produced by the oral administration, for four consecutive days, of three novel HO-1 inducers, 1m, 1a, and 1b, and of DMF. Moreover, our results reveal that these treatments also inhibited the anxiety-and depressive-like behaviors associated with persistent inflammatory pain. Furthermore, all treatments normalized the up-regulation of NLRP3 inflammasome and activated the antioxidant signaling pathway triggered by Nrf2/HO-1 in the paw and/or amygdala.

Our findings revealed, for the first time, the antiallodynic and antihyperalgesic effects, in addition to the reversal of grip strength deficits, produced by the oral administration of 1m, 1a, 1b, and DMF in animals with inflammatory pain. However, while only two days of treatment with 1m was required to inhibit the allodynia, the hyperalgesia, and the grip strength deficits induced by CFA, three days of treatment with 1b, 1a, or DMF were indispensable for the complete inhibition of allodynia. For the full reversion of the hyperalgesia, two days of treatment with 1b and three with 1a or DMF were needed. In addition, whereas the loss of grip strength induced by CFA was completely recovered after two days of treatment with 1b or 1a, four days of DMF treatment were necessary. Therefore, and based on the three tests evaluated in this study, it seems that 1m is the more effective drug in inhibiting the allodynia; 1m and 1b in reducing the hyperalgesia; and 1m, 1a, and 1b in recovering the functional deficits provoked by peripheral inflammation. Thus, this study reveals the higher efficacy of these new HO-1 inducers vs. DMF in inhibiting the nociceptive responses and the grip force deficits provoked by persistent peripheral inflammation, 1m being the most effective.

The analgesic effects produced by 1m, 1a, and 1b, as well as DMF, agreed with previous research revealing the painkiller properties of DMF in animals with arthritis [19]; those produced by another HO-1 inducer, cobalt protoporphyrin IX (CoPP), in mice with CFA-induced inflammatory pain [38,39]; and that made by lentivirus encoding HO-1 in animals with vincristine provoked neuropathic pain [40]. The recovery of functional disabilities produced by 1m, 1a, 1b, and DMF during inflammatory pain agreed with the improvement of the grip strength deficits induced by DMF in animals with the sciatic nerve crushed [23] or with multiple sclerosis [24].

Oxidative stress plays a significant role in the activation of the NLRP3 inflammasome, which has been associated with inflammatory pain [41]. Consistent with previous findings that observed the NLRP3 activation in the spinal cord of animals with CFA-induced inflammatory pain [41], our results furthermore indicated increased levels of this inflammasome in the paw of animals with peripheral inflammation, which might be the main factor responsible for the inflammatory pain. It has also been proven that DMF prevents the priming step of NLRP3 activation [42]. In line with this research, we demonstrated that, in addition to DMF, the new HO-1 inducers, 1m, 1a, and 1b, also reversed the up-regulation of NLRP3 caused by CFA in the paw. This suggests that the analgesia and the retrieval of grip strength produced by 1m, 1a, and 1b might be in part attributed to the normalization of the peripheral overexpression of the NLRP3 inflammasome. Accordingly, a recent study revealed that DMF attenuated arthritic pain by inhibiting the activation of the NLRP3 inflammasome and subsequent expression of caspase-1, IL-1β, and IL-18 in the paw [26].

Previous studies suggested that the activation of the Nrf2 pathway exerts potent analgesic actions in several pain models [13]. Thus, studies involving sulforaphane (SFN), a Nrf2 activator, displayed its painkiller properties in animals with inflammatory pain by enhancing the expression of Nrf2 and of several antioxidant enzymes, including HO-1, in the paw [11]. Additionally, the protein expression of Nrf2 was also upregulated after the oral administration of DMF in the spinal cord of rats with osteoarthritis pain [20]. In accordance with these findings, a significant increase in the Nrf2 levels was observed in the paw of animals treated for four days with DMF, 1m, 1a, and 1b. This shows the capacity of these compounds to induce the expression of this transcription factor as previously demonstrated in silico [29]. Moreover, as expected, an increased expression of HO-1 in the paw of animals with inflammatory pain receiving the oral treatment with DMF, 1m, 1a, and 1b was demonstrated. Additionally, a further increase in HO-1 levels was displayed in animals treated with 1b as compared with DMF, revealing that 1b is more potent than DMF in inducing the HO-1 expression under inflammatory pain conditions. In agreement, a study performed in vitro also demonstrated that 1b increased the HO-1 expression more effectively than DMF [29]. This work also remarked on the high potency of 1m in increasing HO-1 activation, thus explaining the major effectivity of 1m in inhibiting inflammatory pain as compared with 1a, 1b, and DMF. Moreover, and supporting our data, other works reported that DMF inhibited arthritic pain by improving the HO-1 levels in joint tissues [19] and reversed the grip strength loss by increasing the expression of HO-1 in the sciatic nerves [23]. Thus, our results suggest that the analgesic effects and the recovery of grip strength produced by 1m, 1a, and 1b could be attributed, at least in part, to the enhancement of HO-1 expression, together with that of Nrf2, produced by these compounds in the paw.

Nonetheless, we also observed an effect of these drugs in the expression of SOD-1, another antioxidant enzyme activated by Nrf2. That is, the increased SOD-1 levels triggered by peripheral inflammation in the paw were maintained after DMF, 1m, 1a, and 1b treatments. These results coincided with other findings demonstrating high levels of SOD-1 in the joints of arthritic rats treated with DMF [19]. Our data reinforced the antioxidant properties of 1m, 1a, and 1b treatments and further suggested that SOD-1 might also participate in the painkiller and functional actions induced by them during inflammatory pain.

Our data did not show any effects of 1m, 1a, 1b or DMF on the paw levels of NQO1 and GSTM1, two other Nrf2 activated enzymes. In contrast, increased levels of NQO1 were detected in the paws of CFA-injected mice treated with SFN [11]. This shows the differential participation of some antioxidant enzymes in the actions performed by SFN and other HO-1 inducers. Therefore, based on the outcomes of our study, we postulate that treatments with 1m, 1a, 1b, or DMF mainly exert their analgesic and functional effects through inhibiting the local activation of the NLRP3 inflammasome and inducing the peripheral Nrf2/HO-1 and SOD-1 antioxidant pathway.

It is well known that persistent inflammatory pain is associated with several emotional symptoms, such as anxiety and depression [43,44,45,46]. Our results supported these findings, showing that chronic inflammation of the paw, in addition to increasing sensitivity to noxious stimulation and reducing grip strength, also altered the emotive states. Thus, the low visits of the animals to the open arms in the EPM and the low time spent in the central area of the OF tests revealed the anxiety-like symptoms, while the greater immobility time in the TST and FST displayed the depressive-like behaviors associated with chronic inflammatory pain. Interestingly, all these symptoms were reversed by the oral administration of DMF, 1m, 1a, and 1b, for four days, revealing the anxiolytic and antidepressant effects of these compounds under these conditions. In concordance, the administration of SFN also inhibited the susceptibility to develop depression under stress conditions [47] and suppressed the anxiodepressive-like behaviors related to neuropathic pain by normalizing the expression of Nrf2 and HO-1 in the hippocampus and prefrontal cortex [48]. In this study, by evaluating the effects of treatment with DMF, 1m, 1a, and 1b on the expression of several antioxidative proteins in the amygdala, a brain area involved in the control of the emotional components of pain [49], we observed that all drugs increased the HO-1 levels that occurred in the paw. This shows the capacity of the new HO-1 inducers, 1m, 1a and 1b, as well as DMF, orally administered, to induce the expression of HO-1 in both the central and peripheral nervous system. Moreover, and in accordance with the role played by HO-1 in modulating neuropsychiatric disorders [50], the improvement of its expression induced by DMF, 1m, 1a, and 1b treatments in the amygdala might in some way supported their anxiolytic and antidepressant effects during inflammatory pain. Our results also showed, while the expression of Nrf2 and SOD-1 increased in the paws of DMF-, 1m-, 1a-, and 1b-treated mice, no changes in their expression were observed in the amygdala. The lack of changes in the amygdala could probably be related to the fact that, while similar levels of HO-1, NQO1, and GSTM1 were observed in the paws and amygdala of SS–VEH mice, the levels of Nrf2 and SOD-1 in the amygdala of these animals seemed to be higher than in the paws. Therefore, it is possible that the basal high levels of these proteins might be high enough to combat the inflammatory/oxidative responses caused by CFA in this brain area. Nonetheless, additional experiments are required to demonstrate this hypothesis.

It is currently accepted that, besides oxidative stress, inflammation is another of the main mechanisms involved in mood disorders [51,52,53]. Therefore, elevated levels of some inflammatory mediators have been demonstrated in depressive states. Moreover, a link between NLRP3 inflammasome activation and depressive-like behaviors has been shown in animals with lipopolysaccharide-induced inflammation [8]. Considering these factors, we postulated that the up-regulation of NLRP3 induced by CFA in the amygdala supports the role of this inflammasome in the development of the emotional disorders associated with peripheral inflammation. In addition, the normalization of their up-regulation with DMF, 1m, 1a, and 1b treatments suggests that the anxiolytic and antidepressant actions of these compounds could also be mediated by inhibiting NLRP3 activation in the amygdala. The reduced anxiodepressive behaviors associated with diabetes produced by an inhibitor of the NLRP3 inflammasome, MCC950, supports this hypothesis [54]. On the other hand, the fact that stimulation of the Nrf2 pathway reduces inflammation by blocking NLRP3 activation [55] suggests that the modulation of inflammatory pain and associated emotional disorders induced by these new HO-1 inducers could be mediated through inhibiting the inflammatory responses via activation of the antioxidant system.

A limitation of this study could be the fact that we have only evaluated the effects of these treatments in male mice, and, considering the influence of sex on the analgesic effects induced by some treatments, it would have also been interesting to analyze their effects in female mice.

## 5. Conclusions

In summary, this study reported that the oral administration of DMF, 1m, 1a, and 1b produced analgesic effects and reversed the deficits of grip strength provoked by peripheral inflammation in mice, 1m being the most effective. Moreover, these data demonstrated the anxiolytic and antidepressant properties of these compounds during inflammatory pain and proved that their effects might be mainly produced by suppressing the activation of NLRP3 inflammasome in the paw and amygdala and through triggering the Nrf2/HO-1/SOD-1 pathway in the paw and the HO-1 enzyme in the amygdala. These data propose the use of 1m, 1a, 1b, and DMF, especially 1m, as new effective treatments for inflammatory pain and related emotional syndromes.

## Data Availability

Data is contained within the article.

## References

[1] Raja S.N., Carr D.B., Cohen M., Finnerup N.B., Flor H., Gibson S., Keefe F.J., Mogil J.S., Ringkamp M., Sluka K.A. (2020). The revised International Association for the Study of Pain definition of pain: Concepts, challenges, and compromises. Pain.

[2] Mills S.E.E., Nicolson K.P., Smith B.H. (2019). Chronic pain: A review of its epidemiology and associated factors in population-based studies. Br. J. Anaesth..

[3] Callahan L.F., Brooks R.H., Summey J.A., Pincus T. (1987). Quantitative pain assessment for routine care of rheumatoid arthritis patients, using a pain scale based on activities of daily living and a visual analog pain scale. Arthritis Rheum..

[4] Van der Windt D.A., Kuijpers T., Jellema P., van der Heijden G.J., Bouter L.M. (2007). Do psychological factors predict outcome in both low-back pain and shoulder pain?. Ann. Rheum. Dis..

[5] Cohen S.P., Vase L., Hooten W.M. (2021). Chronic pain: An update on burden, best practices, and new advances. Lancet.

[6] Schwan J., Sclafani J., Tawfik V.L. (2019). Chronic Pain Management in the Elderly. Anesthesiol. Clin..

[7] Zhou K., Shi L., Wang Y., Chen S., Zhang J. (2016). Recent Advances of the NLRP3 Inflammasome in Central Nervous System Disorders. J. Immunol. Res..

[8] Arioz B.I., Tastan B., Tarakcioglu E., Tufekci K.U., Olcum M., Ersoy N., Bagriyanik A., Genc K., Genc S. (2019). Melatonin Attenuates LPS-Induced Acute Depressive-Like Behaviors and Microglial NLRP3 Inflammasome Activation through the SIRT1/Nrf2 Pathway. Front. Immunol..

[9] Starobova H., Nadar E.I., Vetter I. (2020). The NLRP3 Inflammasome: Role and Therapeutic Potential in Pain Treatment. Front. Physiol..

[10] Ferrándiz M.L., Nacher-Juan J., Alcaraz M.J. (2018). Nrf2 as a therapeutic target for rheumatic diseases. Biochem. Pharmacol..

[11] Redondo A., Chamorro P.A.F., Riego G., Leánez S., Pol O. (2017). Treatment with Sulforaphane Produces Antinociception and Improves Morphine Effects during Inflammatory Pain in Mice. J. Pharmacol. Exp. Ther..

[12] Wang C., Wang C. (2017). Anti-nociceptive and anti-inflammatory actions of sulforaphane in chronic constriction injury-induced neuropathic pain mice. Inflammopharmacology.

[13] Pol O. (2021). The role of carbon monoxide, heme oxygenase 1, and the Nrf2 transcription factor in the modulation of chronic pain and their interactions with opioids and cannabinoids. Med. Res. Rev..

[14] Cuadrado A., Rojo A.I., Wells G., Hayes J.D., Cousin S.P., Rumsey W.L., Attucks O.C., Franklin S., Levonen A.L., Kensler T.W. (2019). Therapeutic targeting of the NRF2 and KEAP1 partnership in chronic diseases. Nat. Rev. Drug Discov..

[15] Linker R.A., Lee D.H., Ryan S., van Dam A.M., Conrad R., Bista P., Zeng W., Hronowsky X., Buko A., Chollate S. (2011). Fumaric acid esters exert neuroprotective effects in neuroinflammation via activation of the Nrf2 antioxidant pathway. Brain.

[16] Bomprezzi R. (2015). Dimethyl fumarate in the treatment of relapsing-remitting multiple sclerosis: An overview. Ther. Adv. Neurol. Disord..

[17] Majkutewicz I. (2022). Dimethyl fumarate: A review of preclinical efficacy in models of neurodegenerative diseases. Eur. J. Pharmacol..

[18] Chen H., Assmann J.C., Krenz A., Rahman M., Grimm M., Karsten C.M., Köhl J., Offermanns S., Wettschureck N., Schwaninger M. (2014). Hydroxycarboxylic acid receptor 2 mediates dimethyl fumarate’s protective effect in EAE. J. Clin. Investig..

[19] Lal R., Dhaliwal J., Dhaliwal N., Dharavath R.N., Chopra K. (2021). Activation of the Nrf2/HO-1 signaling pathway by dimethyl fumarate ameliorates complete Freund’s adjuvant-induced arthritis in rats. Eur. J. Pharmacol..

[20] Gao S.J., Li D.Y., Liu D.Q., Sun J., Zhang L.Q., Wu J.Y., Song F.H., Zhou Y.Q., Mei W. (2022). Dimethyl Fumarate Attenuates Pain Behaviors in Osteoarthritis Rats via Induction of Nrf2-Mediated Mitochondrial Biogenesis. Mol. Pain.

[21] Singh J., Thapliyal S., Kumar A., Paul P., Kumar N., Bisht M., Naithani M., Rao S., Handu S.S. (2022). Dimethyl Fumarate Ameliorates Paclitaxel-Induced Neuropathic Pain in Rats. Cureus.

[22] Casili G., Lanza M., Filippone A., Cucinotta L., Paterniti I., Repici A., Capra A.P., Cuzzocrea S., Esposito E., Campolo M. (2022). Dimethyl Fumarate (DMF) Alleviated Post-Operative (PO) Pain through the N-Methyl-d-Aspartate (NMDA) Receptors. Antioxidants.

[23] Szepanowski F., Donaldson D.M., Hartung H.P., Mausberg A.K., Kleinschnitz C., Kieseier B.C., Stettner M. (2017). Dimethyl fumarate accelerates peripheral nerve regeneration via activation of the anti-inflammatory and cytoprotective Nrf2/HO-1 signaling pathway. Acta Neuropathol..

[24] Kumar P., Sharma G., Gupta V., Kaur R., Thakur K., Malik R., Kumar A., Kaushal N., Raza K. (2018). Preclinical Explorative Assessment of Dimethyl Fumarate-Based Biocompatible Nanolipoidal Carriers for the Management of Multiple Sclerosis. ACS Chem. Neurosci..

[25] Liu X., Zhou W., Zhang X., Lu P., Du Q., Tao L., Ding Y., Wang Y., Hu R. (2016). Dimethyl fumarate ameliorates dextran sulfate sodium-induced murine experimental colitis by activating Nrf2 and suppressing NLRP3 inflammasome activation. Biochem. Pharmacol..

[26] Cao Y., Hu Y., Jin X.F., Liu Y., Zou J.M. (2023). Dimethyl fumarate attenuates MSU-induced gouty arthritis by inhibiting NLRP3 inflammasome activation and oxidative stress. Eur. Rev. Med. Pharmacol. Sci..

[27] Waza A.A., Hamid Z., Ali S., Bhat S.A., Bhat M.A. (2018). A review on heme oxygenase-1 induction: Is it a necessary evil. Inflamm. Res..

[28] Li X., Clark J.D. (2003). Heme oxygenase type 2 participates in the development of chronic inflammatory and neuropathic pain. J. Pain.

[29] Sorrenti V., Vanella L., Platania C.B.M., Greish K., Bucolo C., Pittalà V., Salerno L. (2020). Novel Heme Oxygenase-1 (HO-1) Inducers Based on Dimethyl Fumarate Structure. Int. J. Mol. Sci..

[30] Pittalà V., Vanella L., Maria Platania C.B., Salerno L., Raffaele M., Amata E., Marrazzo A., Floresta G., Romeo G., Greish K. (2019). Synthesis, in vitro and in silico studies of HO-1 inducers and lung antifibrotic agents. Future Med. Chem..

[31] Chaplan S.R., Bach F.W., Pogrel J.W., Chung J.M., Yaksh T.L. (1994). Quantitative assessment of tactile allodynia in the rat paw. J. Neurosci. Methods.

[32] Hargreaves K., Dubner R., Brown F., Flores C., Joris J. (1988). A new and sensitive method for measuring thermal nociception in cutaneous hyperalgesia. Pain.

[33] Montilla-García Á., Tejada M.Á., Perazzoli G., Entrena J.M., Portillo-Salido E., Fernández-Segura E., Cañizares F.J., Cobos E.J. (2017). Grip strength in mice with joint inflammation: A rheumatology function test sensitive to pain and analgesia. Neuropharmacology.

[34] Walf A.A., Frye C.A. (2007). The use of the elevated plus maze as an assay of anxiety-related behavior in rodents. Nat. Protoc..

[35] Kraeuter A.K., Guest P.C., Sarnyai Z. (2019). The Open Field Test for Measuring Locomotor Activity and Anxiety-like Behavior. Methods Mol. Biol..

[36] Steru L., Chermat R., Thierry B., Simon P. (1985). The tail suspension test: A new method for screening antidepressants in mice. Psychopharmacology.

[37] Porsolt R.D., Le Pichon M., Jalfre M. (1977). Depression: A new animal model sensitive to antidepressant treatments. Nature.

[38] Moreno P., Cazuza R.A., Mendes-Gomes J., Díaz A.F., Polo S., Leánez S., Leite-Panissi C.R.A., Pol O. (2019). The Effects of Cobalt Protoporphyrin IX and Tricarbonyldichlororuthenium (II) Dimer Treatments and Its Interaction with Nitric Oxide in the Locus Coeruleus of Mice with Peripheral Inflammation. Int. J. Mol. Sci..

[39] Cazuza R.A., Batallé G., Bai X., Leite-Panissi C.R.A., Pol O. (2022). Effects of treatment with a carbon monoxide donor and an activator of heme oxygenase 1 on the nociceptive, apoptotic and/or oxidative alterations induced by persistent inflammatory pain in the central nervous system of mice. Brain Res. Bull..

[40] Shen Y., Zhang Z.J., Zhu M.D., Jiang B.C., Yang T., Gao Y.J. (2015). Exogenous induction of HO-1 alleviates vincristine-induced neuropathic pain by reducing spinal glial activation in mice. Neurobiol. Dis..

[41] Hua T., Wang H., Fan X., An N., Li J., Song H., Kong E., Li Y., Yuan H. (2022). BRD4 Inhibition Attenuates Inflammatory Pain by Ameliorating NLRP3 Inflammasome-Induced Pyroptosis. Front. Immunol..

[42] Tastan B., Arioz B.I., Tufekci K.U., Tarakcioglu E., Gonul C.P., Genc K., Genc S. (2021). Dimethyl Fumarate Alleviates NLRP3 Inflammasome Activation in Microglia and Sickness Behavior in LPS-Challenged Mice. Front. Immunol..

[43] Narita M., Kaneko C., Miyoshi K., Nagumo Y., Kuzumaki N., Nakajima M., Nanjo K., Matsuzawa K., Yamazaki M., Suzuki T. (2006). Chronic pain induces anxiety with concomitant changes in opioidergic function in the amygdala. Neuropsychopharmacology.

[44] Parent A.J., Beaudet N., Beaudry H., Bergeron J., Bérubé P., Drolet G., Sarret P., Gendron L. (2012). Increased anxiety-like behaviors in rats experiencing chronic inflammatory pain. Behav. Brain Res..

[45] Huang H.Y., Liao H.Y., Lin Y.W. (2020). Effects and Mechanisms of Electroacupuncture on Chronic Inflammatory Pain and Depression Comorbidity in Mice. Evid. Based Complement. Altern. Med..

[46] Guan S.Y., Zhang K., Wang X.S., Yang L., Feng B., Tian D.D., Gao M.R., Liu S.B., Liu A., Zhao M.G. (2020). Anxiolytic effects of polydatin through the blockade of neuroinflammation in a chronic pain mouse model. Mol. Pain.

[47] Dang R., Guo Y.Y., Zhang K., Jiang P., Zhao M.G. (2019). Predictable chronic mild stress promotes recovery from LPS-induced depression. Mol. Brain.

[48] Ferreira-Chamorro P., Redondo A., Riego G., Leánez S., Pol O. (2018). Sulforaphane Inhibited the Nociceptive Responses, Anxiety- and Depressive-Like Behaviors Associated With Neuropathic Pain and Improved the Anti-allodynic Effects of Morphine in Mice. Front. Pharmacol..

[49] Neugebauer V., Mazzitelli M., Cragg B., Ji G., Navratilova E., Porreca F. (2020). Amygdala, neuropeptides, and chronic pain-related affective behaviors. Neuropharmacology.

[50] Neis V.B., Rosa P.B., Moretti M., Rodrigues A.L.S. (2018). Involvement of Heme Oxygenase-1 in Neuropsychiatric and Neurodegenerative Diseases. Curr. Pharm. Des..

[51] Siwek M., Sowa-Kućma M., Dudek D., Styczeń K., Szewczyk B., Kotarska K., Misztakk P., Pilc A., Wolak M., Nowak G. (2013). Oxidative stress markers in affective disorders. Pharmacol. Rep..

[52] Hodes G.E., Kana V., Menard C., Merad M., Russo S.J. (2015). Neuroimmune mechanisms of depression. Nat. Neurosci..

[53] Harsanyi S., Kupcova I., Danisovic L., Klein M. (2022). Selected Biomarkers of Depression: What Are the Effects of Cytokines and Inflammation?. Int. J. Mol. Sci..

[54] Zhai Y., Meng X., Ye T., Xie W., Sun G., Sun X. (2018). Inhibiting the NLRP3 Inflammasome Activation with MCC950 Ameliorates Diabetic Encephalopathy in db/db Mice. Molecules.

[55] An Y.W., Jhang K.A., Woo S.Y., Kang J.L., Chong Y.H. (2016). Sulforaphane exerts its anti-inflammatory effect against amyloid-β peptide via STAT-1 dephosphorylation and activation of Nrf2/HO-1 cascade in human THP-1 macrophages. Neurobiol. Aging.

